# A Case of Severe Burn Treated by White Paint

**Published:** 1859-12

**Authors:** O. C. Gibbs

**Affiliations:** Frewsburg, Chautauque County, New York


ARTICLE III.

### A CASE OF SEVERE BURN, TREATED BY WHITE PAINT.—RECOVERY, ETC.

BY O. C. GIBBS, M.D., FREWSBURG, CHAUTAUQUE CO., N. Y.

April 29th, 1858, was called to S. B., a female child, aged 11 years, in consultation with Dr. Hazeltine. The girl's clothes had taken fire while she was in the house alone. She ran into the street, but soon fell, exhausted, before help came to her aid. The whole back, from the hips to the shoulders, a surface thirteen inches wide and fifteen inches long, was completely charred. The back of both arms, from the elbows to the shoulders, was also burned in the same manner.

Being away on business, I did not see the case until after dressings of lint and sweet oil were applied. I saw no more of the case until five days later, when I was called to it. I found the patient in complete prostration, and laboring under painful strangury.

I urged the friends to send for the attending physician, which they utterly refused to do, and I consequently assumed the responsibilities of the case. The dressings were removed, and, on cutting through the charred flesh, I found the texture destroyed to the depth of at least half an inch. White paint was applied over the whole extent of the burned surface, and quinine and opium, with brandy, was administered internally. The dressings were changed no sooner than the circumstances of the case seemed to render necessary.

On the ninth day, the suppurative discharge became fully established. The bed was covered with oiled silk, and she chose to lay upon the burned surfaces rather than in any other position. The discharges were profuse and offensive, and the patient seemed to lay in a trough of putrefaction. Whenever new dressings were applied, the sufferings were so acute that the patient would shriek most piteously, and earnestly and beseechingly urge that we put an end to her sufferings by death.

Elixir vitriol was occasionally given, with the hope of preventing the poisonous action of the lead; and the dressings, the bed and the floor were freely sprinkled with a solution of the chlorate of lime. This treatment was continued for several weeks, and under the profuse purulent and exhausting discharges, the patient wasted away to almost a skeleton.

The treatment was continued for several months, with only slight variations. Sometimes the white paint dressings were discontinued, and white lead rubbed up with lard was used instead. This substitution was made more with reference to satisfying the friends with the idea of change, rather than because of any expected additional advantage to be derived from it. The elixir vitriol was given only for a short time; and when the exhausting discharges had considerably diminished, all stimulant and tonic medicines were abandoned.

The treatment, indicated above, was pursued for the first six months. After this, at each dressing, the surface of the suppurating sore was freely sprinkled with tannin, and the whole covered with an ointment made in the following manner:

| | | |
|---|---|---|
| ℞ | Sugar of lead, | $\frac{1}{4}$ lb. |
| | Linseed oil, | 1 pint. |
| | Suet, a sufficient quantity. | |

Dissolve the lead in the oil, by the aid of heat, and add suet sufficient to bring to the consistence of an ointment, stirring until cold.

The tannin and the ointment were continued until the sore was entirely healed, which occurred at about the end of the tenth month from the date of the accident. The patient is now quite fleshy and in good health.

Many have feared to make use of lead applications to suppurating surfaces, for fear of poisoning from its absorption.

The main point of interest in this case is the protracted use both of the carbonate and the acetate of lead to a very large surface of the body, with no evidence of any absorption of that agent.

The fact that the granulations continued healthy through so long a period, and that so extensive destruction of integument was so kindly and entirely replaced, is sufficient evidence of the propriety of the applications.

There is one point in this case to which I wish to direct attention. The friends were directed not to wash the sore at all. Through the repeated importunities of neighbors, an attempt was made to use soap and water, in cleansing the sore, at one time in my absence, perhaps about three months after the receipt of the injury. The pain it occasioned was extreme, and the sores inflamed considerably, and did not recover their former state for several days. Strange as it may seem, healthy pus is the best possible protection to healthy granulations, and the best security against their unhealthiness.

When new dressings were applied, the sore was quickly and very gently wiped with dry lint only. So fast as new skin was formed, an attempt was made to give it firmness, by occasionally sprinkling it with tannin. The amount of purulent discharge was very great, and the patient's recovery was certainly unexpected.

The proper treatment of burns is becoming a matter of increasing interest, in these days of *hoops*, when accidents from clothes taking fire is of frequent occurrence.

